# Improved localization precision via restricting confined biomolecule stochastic motion in single-molecule localization microscopy

**DOI:** 10.1515/nanoph-2021-0481

**Published:** 2021-11-16

**Authors:** Jielei Ni, Bo Cao, Gang Niu, Danni Chen, Guotao Liang, Tingying Xia, Heng Li, Chen Xu, Jingyu Wang, Wanlong Zhang, Yilin Zhang, Xiaocong Yuan, Yanxiang Ni

**Affiliations:** Nanophotonics Research Center, Shenzhen Key Laboratory of Micro-Scale Optical Information Technology and Institute of Microscale Optoelectronics, College of Physics and Optoelectronic Engineering, College of Electronics and Information Engineering, Shenzhen University, Shenzhen 518060, China; Dr. Neon Technology Ltd, Shenzhen, 518060, China; Phil Rivers Technology, Beijing 100871, China; Joint Turing-Darwin Laboratory of Phil Rivers Technology Ltd. and Institute of Computing Technology, Chinese Academy of Sciences, Beijing 100190, China; Western Institute of Computing Technology, Chinese Academy of Science, Chongqing 400000, China; Hefei National Laboratory for Physical Sciences at Microscale, School of Basic Medical Sciences, Division of Life Sciences and Medicine, University of Science and Technology of China, Hefei 231299, China; Tsinghua-Berkeley Shenzhen Institute (TBSI), Tsinghua University, Shenzhen 518055, China

**Keywords:** confined stochastic motion, localization precision, single-molecule localization microscopy

## Abstract

Single-molecule localization microscopy (SMLM) plays an irreplaceable role in biological studies, in which nanometer-sized biomolecules are hardly to be resolved due to diffraction limit unless being stochastically activated and accurately located by SMLM. For biological samples preimmobilized for SMLM, most biomolecules are cross-linked and constrained at their immobilizing sites but still expected to undergo confined stochastic motion in regard to their nanometer sizes. However, few lines of direct evidence have been reported about the detectability and influence of confined biomolecule stochastic motion on localization precision in SMLM. Here, we access the potential stochastic motion for each immobilized single biomolecule by calculating the displacements between any two of its localizations at different frames during sequential imaging of Alexa Fluor-647-conjugated oligonucleotides. For most molecules, localization displacements are remarkably larger at random frame intervals than at shortest intervals even after sample drift correction, increase with interval times and then saturate, showing that biomolecule stochastic motion is detected and confined around the immobilizing sizes in SMLM. Moreover, localization precision is inversely proportional to confined biomolecule stochastic motion, whereas it can be deteriorated or improved by enlarging the biomolecules or adding a post-crosslinking step, respectively. Consistently, post-crosslinking of cell samples sparsely stained for tubulin proteins results in a better localization precision. Overall, this study reveals that confined stochastic motion of immobilized biomolecules worsens localization precision in SMLM, and improved localization precision can be achieved via restricting such a motion.

## 1 Introduction

Single-molecule localization microscopy (SMLM), such as stochastic optical reconstruction microscopy (STORM) [1], direct STORM (dSTORM) [2, 3], photoactivation localization microscopy (PALM) [4], and fluorescence PALM (FPALM) [5] has circumvented the diffraction barrier and offered lateral resolution of 15–25 nm [1], [2], [3], [4], [5], [6]. Due to the unmatched high resolution among all fluorescence microscopies and its noninvasive property to biological samples, SMLM is irreplaceable in resolving single molecules in biological studies, one important aim for which is tovisualize nanoscale-sized biomolecules *in situ* including proteins, DNA, and lipids in cells [7, 8].

SMLM provides super-resolution via stochastically activating photo-switchable fluorophores and accurately determining their localizations. Biomolecules of interest are commonly labeled with switchable fluorophores via covalent conjugation [2, 9] or genetically encoding protein fusion [10, 11] for high resolution visualization. To maintain intracellular ultra-structures, biological samples are commonly preimmobilized via cross-linking at the beginning of sample preparation, so that most biomolecules cannot diffuse but retain at their original positions except some transmembrane and membrane-bound proteins that require special reagents for immobilization [12, 13]. The precision in localizing single molecules in SMLM is crucial for image resolution and mainly determined by the number of photons collected from the activating fluorophores, such as Alexa Fluor-647 (AF647) [14], [15], [16]. However, in light of the high resolution, any movement of the biomolecules during acquisition probably increases localization uncertainty. In this regard, intense studies have been focused on sample drift correction to ensure the high image quality [17], [18], [19]. Since endogenous biomolecules as well as the antibodies or probes binding to them exhibit sizes of tens of nanometers [2, 9, 20], confined stochastic motion of immobilized biomolecules is expected to be detected in SMLM and result in increased localization uncertainty. However, there are so far few lines of evidence about whether confined biomolecule stochastic motion can be detected or affect localization precision in SMLM.

Studies of confined biomolecule stochastic motion in SMLM have been limited for several reasons. Firstly, the stochastic activation of fluorophores offers discontinuous trajectory that is far insufficient for tracking the biomolecule motion. Secondly, the range of finite regions for confined stochastic motion of immobilize biomolecules are comparable to the spatial resolution of SMLM, which makes it challenging to identify stochastic motion. Thirdly, due to dense labeling, it is almost impossible to identify stochastic motion of biomolecules in regular biological samples. Therefore, new analysis is needed to systematically clarify the detectability and potential impact of immobilized biomolecules in SMLM, so that effective strategies could be applied to improve image resolution.

To address this issue, we applied here single-molecule samples containing sparsely-immobilized AF647-conjugated oligonucleotides, which act as a quantifiable model for systematically assessing confined biomolecule stochastic motion. These oligomers are of 12–30 nm in sizes, which are within the regular range of biomolecule sizes. We evaluated the potential stochastic motion of immobilized molecules based on data collected from dSTORM imaging of these single-molecule samples. Briefly, we calculated the displacements between two localizations of each molecule that has been stochastically activated and captured at different time points during the sequential imaging process. Z_1_-score was proposed for each molecule to statistically evaluate the significance of localization displacements at shortest time intervals (between temporally neighboring camera frames) compared to those at random interval (between two random frames). It turned out that, for most of the molecules examined, the z_1_-score is negative even after sample drift correction, showing that stochastic motion is detected for immobilized molecules during sequential imaging. In regard to different frame intervals, the localization displacements were observed to increase with intervals and then saturate, suggesting stochastic motion of biomolecules is confined within a finite region. Furthermore, localization precision was found to be inversely proportional to the amplitude of molecule stochastic motion. Moreover, we revealed that enlarging molecule sizes or adding an extra step of postcrosslinking at the end of sample preparation decreased or improved localization precision, respectively, which implies strategies for optimizing localization precision and image resolution via minimizing molecule stochastic motion in SMLM.

## 2 Results

### 2.1 Confined stochastic motion of immobilized single biomolecules

**Figure 1:**
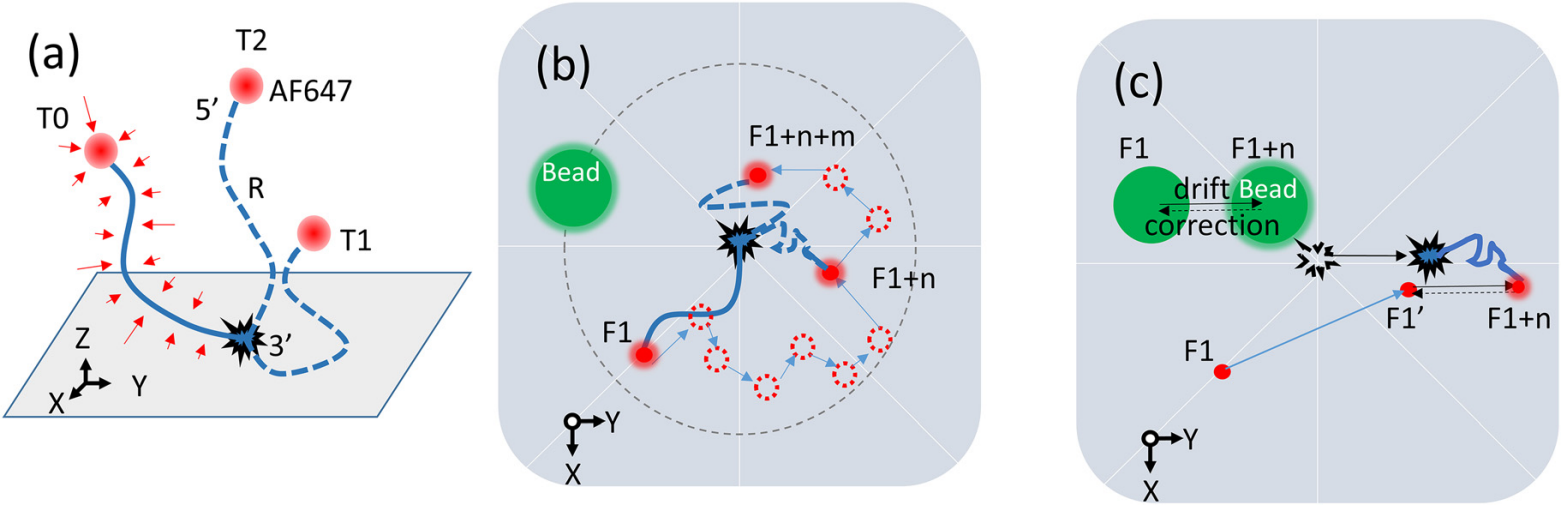
Illustration of confined stochastic motion of immobilized oligonucleotides in sequential imaging. (a) The 40-nucleotide oligomers, each of which is conjugated to a single AF647 (red dot) at 5′ end and to a biotin at 3′ end, are sparsely immobilized on gelatin-coated glass surface via biotin nonspecific binding. These oligonucleotide molecules are expected to exhibit sizes of around 12–20 nm (R), which are within the size range of most biomolecules in regular cell samples for STORM. Given the nanometer sizes, each oligonucleotide is subjected to random collisions (red arrows) from surrounding solution molecules and consequently undergoes confined stochastic motion with one end fixed at its immobilized site (black star). In this regard, each molecule in imaging solution keeps continuously and irregularly moving within a constrained cycle region, which takes the immobilized site as center and R as radius (gray dashed line). During the acquisition, the AF647 on each biomolecule would be stochastically activated, and its position can be recorded for determining localization of the entire biomolecule at different time points (e.g. T0, T1, T2, a), which are corresponding to different frames (e.g. F1, F1 + *n*, F1 + *n* + *m*) in an image sequence (b). (c) Sample drift occurring during sequential imaging can be corrected with the use of fixed fluorescence bead that remains fluorescent during the entire experiment (filled green cycle). For example, the sample shifts during F1 to F1 + *n* (black solid arrow) can be corrected by comparing the position of bead at F1 and F1 + *n* (black dashed arrow). However, for the biomolecule, its position change is actually a combined effect of sample drift (black arrow) and confined stochastic motion (blue arrow), and its corrected localization (F1 + *n*’) is different from the original position at F1. AF647; F1, frame (1); F1 + *n*, frame (1 + *n*); F1 + *n* + *m*, frame (1 + *n* + *m*); SM, stochastic motion.

To assess the potential stochastic motion of immobilized biomolecules in SMLM, we applied in this study single-molecule samples containing 40-nucleotide oligomers, which are 12–20 nm in sizes, to mimic regular biomolecules, including endogenous molecules and their linkers like antibodies or probes in biological samples. Each oligonucleotide is conjugated to an AF647 molecule at 5′ end for reporting its position and sparsely immobilized on glass (Figure 1(a)). We chose to immobilize these oligonucleotides via nonspecific binding of biotin at their 3′ ends to gelatin-coated glass rather than specifically tethering these molecules to streptavidin-coated glass via biotin-streptavidin association, because a tetrameric protein streptavidin capable of binding to four biotin molecules will make it impossible to achieve position coordinates of single-fluorophores. In light of their nanometer sizes, we hypothesized that AF647-conjugated oligonucleotides in imaging solution stochastically move around the immobilized sites within a confined region cycled and that the stochastic movement can be recognized by SMLM and probably deteriorate localization precision. Due to the stochastic activation of fluorophore, molecule motion indicated by the AF647 (red dashed circle) is randomly recorded in different frames (F1, F1 + *n*, F1 + *n* + *m*, Figure 1(b)), which provides insufficient information for outlining the biomolecule trajectory. In regard to sample drift occurring at an interval of *n* frames, for example between F1 and F1 + *n*, it can be corrected back to its original position at F1 with the reference of the fluorescent bead that continuously emits photons during the acquisition (Figure 1(c), black dashed arrow). Meanwhile, for biomolecule undergoing confined stochastic motion (blue arrow) besides sample drifts (black solid arrow), its localization after correction is a new position (marked as F1 + *n*′) instead of the original position at F1, because the stochastic motion cannot be corrected by drift compensation. Therefore, localizations of each single biomolecule during sequential imaging can provides essential clues for assessing stochastic motion.

### 2.2 Using Z-score to study confined stochastic motion

**Figure 2:**
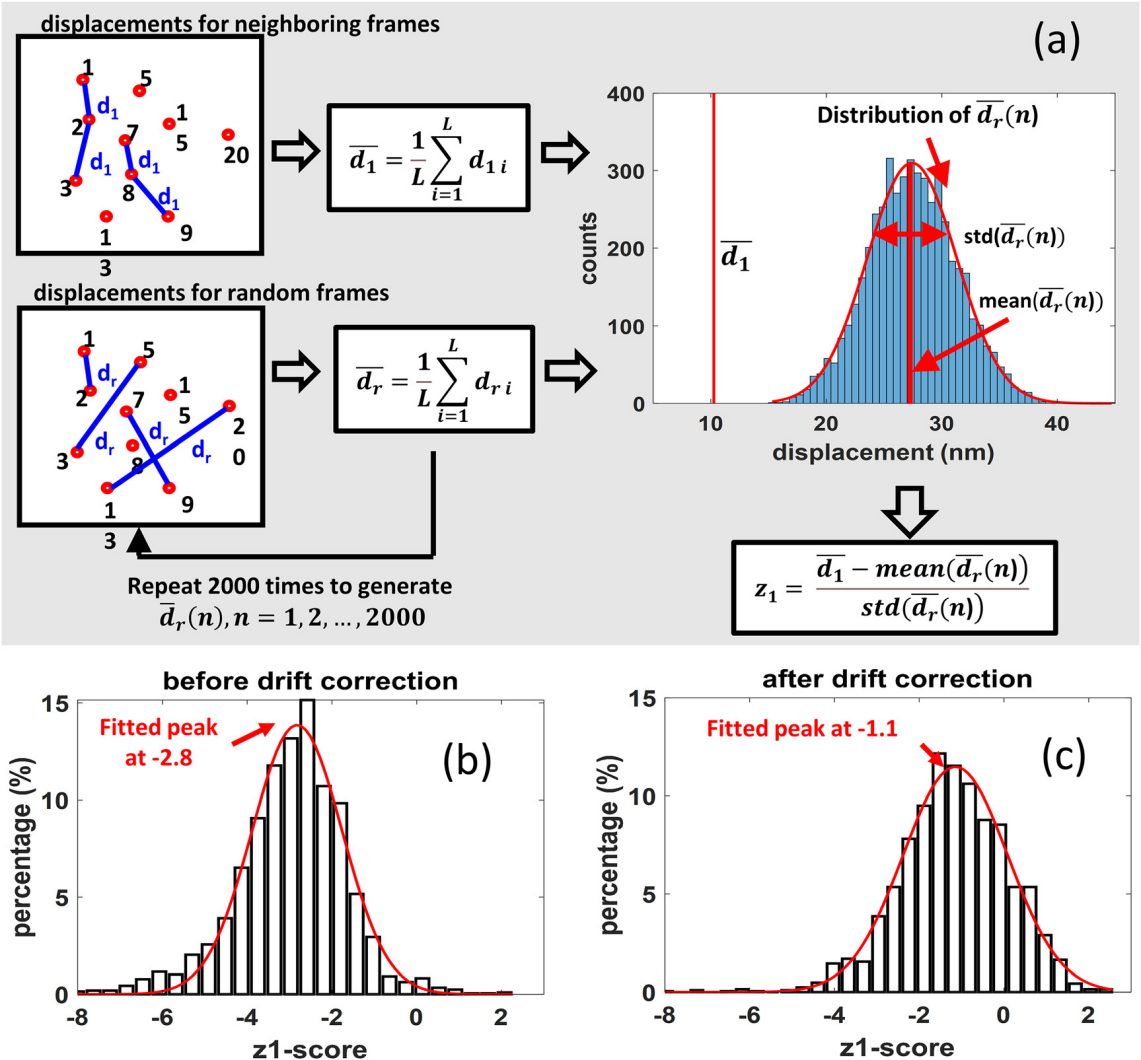
Calculation of neighboring z-scores of immobilized biomolecules shows the existence of biomolecule stochastic motion after drift correction. (a) Illustration of neighboring z-score ( $z_{1}$ -score) algorithm for a certain molecule. Left-top inset illustrates the displacements $d_{1}$ for temporally neighboring localizations. Localizations of a molecule are represented by red circles with frame numbers, from which contain L pairs of neighboring frames ( L=4 in this example). The displacements $d_{1}$ are calculated from the L pairs and depicted as blue lines; Left-bottom inset shows one random sampling of localizations with the same number of pairs (sample size, L ) to calculate $d_{r}$ (depicted as blue line). The random sampling is repeated 2000 times, each time generating one mean value of $d_{r}$ . The histogram of the 2000 $\bar{d_{r}}$ is plotted in the right inset, which shows a normal distribution. The mean value of $d_{1}$ is also plotted as the red line for comparison. The $z_{1}$ -score determines whether the mean of two sampling groups are statistically significant to each other. (b) and (c) The distribution of $z_{1}$ -scores of 2073 molecules, showing a peak at a low value −2.8 before drift correction (b) or at −1.1 after correction (c), respectively.

To address if stochastic motion of immobilized biomolecules can be detected in SMLM, we collected imaging data from dSTORM experiments with single molecule samples and calculated for each single molecule the displacements between any two of its localizations during sequential imaging. Then, $z_{1}$ -score was used for each single molecule to assess the significance of the localization displacements between two temporally neighboring frames compared to those between two random frames. The calculation of $z_{1}$ -score is illustrated in Figure 2(a).

For each single fluorophore, we defined its localizations as $Loc_{i}\left(x_{i},y_{i},f_{i}\right),i=1,2,3,\ldots ,N$ , where N is the total number of localizations in the 8000 frame-sequence and $f_{i}$ is the frame number. The displacement for two localizations in temporally neighboring frames in the dataset, which is denoted as $d_{1}$ ,

(1) $$d_{1}=\sqrt{{\left(x_{i+1}-x_{i}\right)}^{2}+{\left(y_{i+1}-y_{i}\right)}^{2}},if\;f_{i+1}-f_{i}=1.$$

The mean value of all $d_{1}$ in the entire dataset {Loc} can be calculated as

(2) $$\bar{d_{1}}=mean\left(d_{1i}\right)=\frac{1}{L}\overset{L}{\underset{i=1}{\sum }}d_{1i}$$

Similarly, the displacement between two localizations in random frames of an image sequence, which is denoted as $d_{r}$ , can be calculated as,

(3) $$d_{r}=\sqrt{{\left(x_{i}-x_{j}\right)}^{2}+{\left(y_{i}-y_{j}\right)}^{2}},i,j\;\epsilon \;\left\{Loc\right\}$$

To statistically compare $d_{1}\text{ and }d_{\text{r}}$ for each molecule, we took 2000 random samples from its data set {Loc} with the same sample size ( L ) as that in the calculation of $d_{1}$ , and calculated the mean of $d_{r}$ for each random sample as (Figure 2(a), left),

(4) $$\bar{d_{r}}=mean\left(d_{ri}\right)=\frac{1}{L}\overset{L}{\underset{i=1}{\sum }}d_{ri}$$

According to Central-Limit Theorem, the distribution of the 2000 sample means would be a normal distribution, as illustrated in the inset (right top) of Figure 2(a).

To evaluate the statistical difference between $d_{1}$ and $d_{r}$ for a certain molecule, we defined the $z_{1}$ -score for localizations from temporally neighboring frames as “neighboring z-score”, denoted as $z_{1}$ by

(5) $$z_{1}=\frac{\bar{d_{1}}-mean\left(\bar{{\text{d}}_{r}}\left(\text{n}\right)\right)}{std\left(\bar{d_{r}}\left(\text{n}\right)\right)}$$

The $z_{1}$ -score defines how many standard deviations between the neighboring localization displacements and the random localization displacements for a certain molecule. A negative $z_{1}$ -score reflects that the $\bar{d_{1}}$ is smaller than the mean of $\bar{d_{r}}$ , which means that the localizations of a certain molecule in an image sequence spread with increasing time interval and implies molecule motion during acquisition. To assess the potential stochastic movement of immobilized biomolecules during image acquisition, we calculated the $z_{1}$ -scores both before and after drift correction for 2073 oligonucleotide molecules examined by dSTORM imaging. It was found that the $z_{1}$ -scores of most molecules situate near the peak of −2.8 before correction (Figure 2(b)) and remarkably increase by 1.7 in sample drift correction (Figure 2(c)), suggesting effective drift correction. However, the main molecule fraction is still at a negative $z_{1}$ -score of around −1.1 after correction, instead of zero (Figure 2(c)). These results showed that the molecules are affected not only by sample drift but also by some other motion, like stochastic motion, which cannot be corrected by the fluorescent bead.

To understand the molecule motion except sample drift, we further analyzed the dynamics of the different z-scores of each molecule at different frame intervals. The displacement for two localizations at an interval of m frames in the dataset, which is denoted as $d_{\text{m}}$ , is calculated by revising Eq. (1) to

(6) $$d_{m}=\sqrt{{\left(x_{k}-x_{i}\right)}^{2}+{\left(y_{k}-y_{i}\right)}^{2}},if\;f_{k}-f_{i}=m.$$

The corresponding z-score, which we defined as “dynamic z-score” and denoted as $z_{m}$ , is calculated as

(7) $$z_{m}=\frac{\bar{d_{m}}-mean\left(\bar{d_{r}}\left(\text{n}\right)\right)}{std\left(\bar{d_{r}}\left(\text{n}\right)\right)}$$

**Figure 3:**
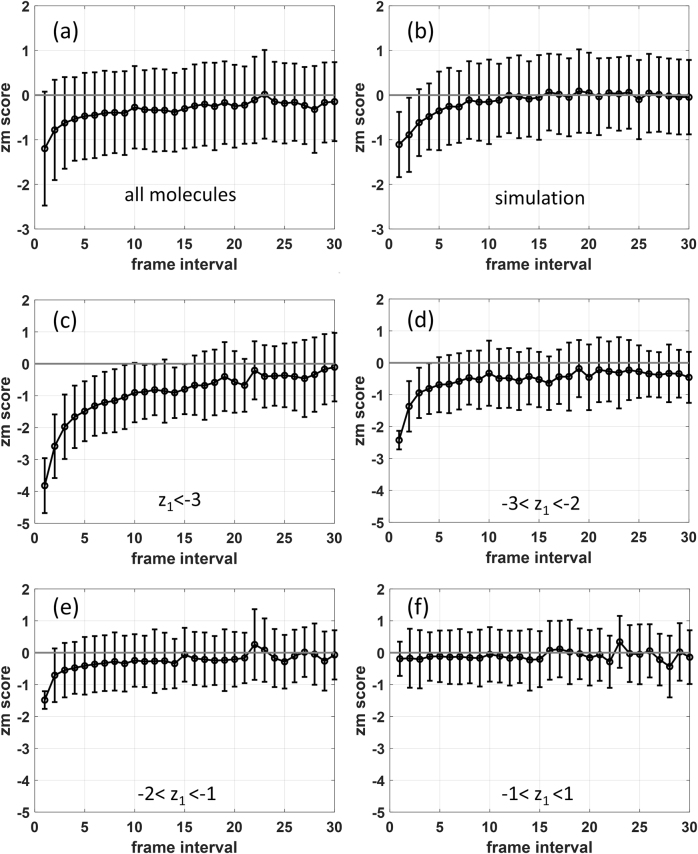
Experimental and theoretical calculations of the dynamic $z_{m}$ -score after drift correction show the stochastic motion of immobilized bio-molecules within confined regions. (a) The $z_{m}$ -scores were plotted based on STORM imaging data of 2073 single molecules when frame interval m increasing from 1 to 30. Error bar represents the standard deviation of $z_{m}$ -scores of 2073 molecules for a certain interval number m. (b) The simulated $z_{m}$ -scores averaged by 200 molecules show similar saturation pattern when frame interval m increasing from 1 to 30. (c)–(f) The total 2073 molecules are divided into four groups according to different scales of z_1_-scores, and their corresponding $z_{m}$ -scores are separately plotted. For the molecules with a z_1_ < −1 (c)–(e), the $z_{m}$ -scores increase and saturate at around 0 after m reaches a certain number. For the molecules with a $z_{1}$ -scores between −1 and 1 (f), the $z_{m}$ -scores randomly fluctuate around zero.

Figure 3(a) shows the ensemble average of $z_{m}$ -scores over 2073 molecules from our dSTORM experiment with frame interval m increasing from 1 to 30. The dynamic curve starts from the ensemble average of $z_{1}$ with a value of −1.1, then increases with frame interval, and finally saturates at around zero after a certain interval. We speculated such a pattern to origin from molecule stochastic motion within a finite region whose boundary is determined by the length of oligonucleotides. At small frame interval, the ensemble averaged displacement increases with time. After a certain time interval when the movement reaches to the entire area, the displacement is restricted by the boundary.

To further confirm our speculation, we simulated the random walk of 200 molecules within a confined circular region with radius within the range of 12–30 nm, which is set as 14.6 nm in our simulation. In light of their nanometer sizes, biomolecules are expected to be subject to random impulses due to frequent collisions by surrounding liquid molecules and to move continuously but irregularly. Meanwhile, since the biomolecules are immobilized, there is a force restricting the movement within confined area. The details of the simulation are described in **Materials and methods**. The simulation result was depicted in Figure 3(b). The ensemble average of $z_{m}$ -scores starts at −1.1, which is in good agreement with the $z_{1}$ value of the experimental curve in Figure 3(a). With increasing frame interval, the $z_{m}$ -score gradually increases and finally reaches zero. In the experimental curve in Figure 3(a), the $z_{m}$ -score finally saturates to a value below zero, which deviates from the simulation curve and probably can be attributed to residue drift in the experiment.

Although the molecules are subjected to the same imaging buffer and with the same molecule size, due to the random feature of forces by collisions, for different molecules, the motion over a frame interval varies in magnitude and direction. Besides, due to sparse activation, the molecule fluoresces only for a few frames. At different frames, the positions it reported would show different degree of aggregation. As a result, the values of $d_{r}$ would vary for different molecules. A low $z_{1}$ -score suggests the molecule having either small $d_{1}$ or large $d_{r}$ . In Figure 3(c)–(f), 2073 molecules from our experiment were grouped according to their $z_{1}$ -score, and the dynamics of $z_{m}$ -score of each group were demonstrated. We can see that molecules with lower $z_{1}$ -score would reach to saturation with a longer frame interval. In Figure 3(f), the $z_{m}$ -scores show no dependence on frame interval, indicating the difference between $d_{1}$ and $d_{r}$ cannot be detected by our system.

### 2.3 Confined stochastic motion worsens localization precision

**Figure 4:**
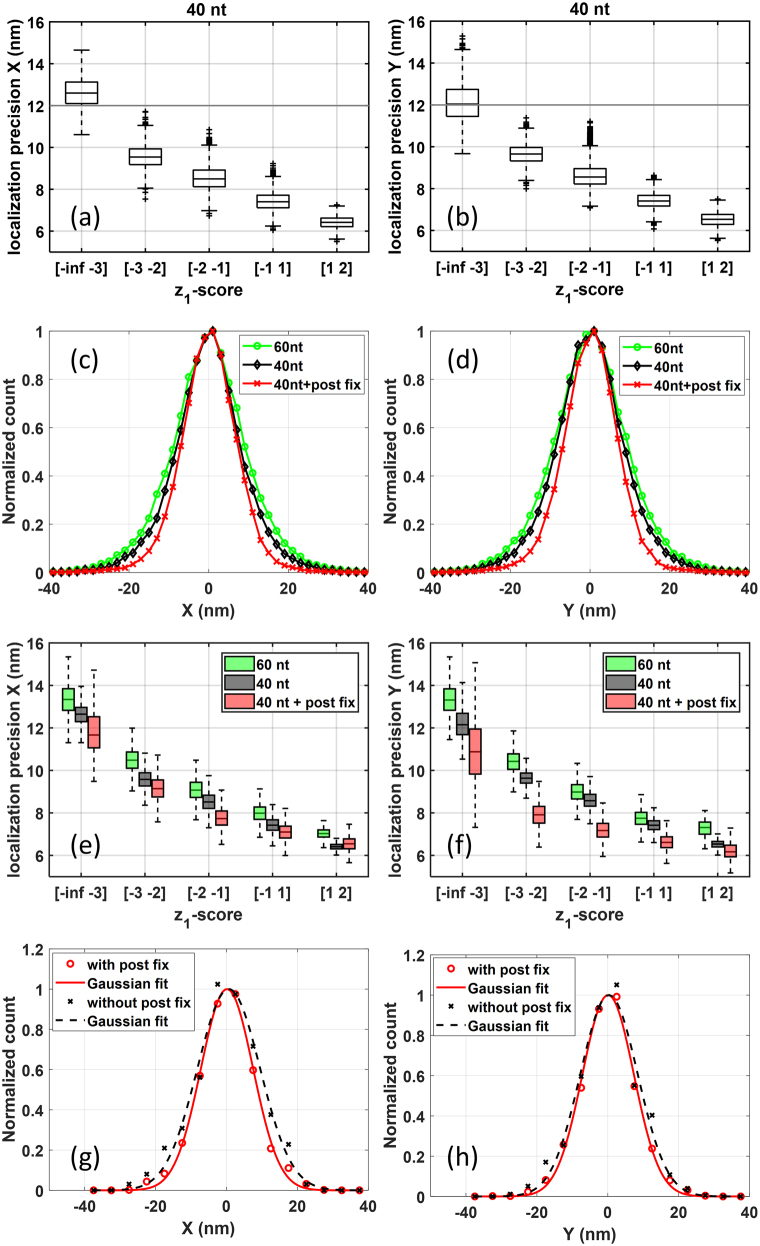
Localization precision in x-direction (left panel) and y-direction (right panel) are affected by the stochastic motion of bio-molecules. (a) and (b) Boxplot of localization precision distribution calculated from molecules with different $z_{1}$ -scores. Within each box, horizontal black lines denote median values; boxes extend from the 25th to the 75th percentile of each group’s distribution of values; vertical extending lines denote adjacent values (i.e., the most extreme values within 1.5 interquartile range of the 25th and 75th percentile of each group); (c) and (d) Comparison of the normalized histogram of localizations from 40 nt oligonucleotides (black line with diamond markers), 60 nt oligonucleotides (green line with circle markers) and post-fixed 40 nt oligonucleotides (red line with cross markers); (e) and (f) comparison of localization precision of similar $z_{1}$ -scores from 40 nt oligonucleotides (black), 60 nt oligonucleotides (green) and post-fixed 40 nt oligonucleotides (red); (g) and (h) Comparison of normalized histogram of localizations from 40 nt oligonucleotides with (red circle) and without (black cross) post fix. The red line shows the Gaussian fit of the experimental data (red circle) from 40 nt oligonucleotides with post fix. The black line shows the Gaussian fit of the experimental data (black cross) from 40 nt oligonucleotides without post fix.

Due to the high resolution and long acquisition time of dSTORM, we suspect that molecule stochastic motion would lead to higher localization uncertainty. To analyse the relation between molecule motion and localization precision, molecules from our dSTORM experiments are grouped according to different $z_{1}$ -scores for calculating the localization precision. In this calculation, molecules were firstly grouped by their $z_{1}$ -scores: Group I with 146 molecules with $z_{1}<-3$ , Group II with 364 molecules with $-3<z_{1}<-2$ , Group III with 651 molecules with $-2<z_{1}<-1$ , Group IV with 839 molecules with $-1<z_{1}<1$ , Group V with 68 molecules with $1<z_{1}<2$ . To ensure the localization precision for each group were calculated from the same number of molecules, random sampling was performed in this calculation. We take 2000 samples for each group, all of the same size of 30 molecules, and compute the localization precision of each sample. The localization precision was calculated as the standard deviation of localizations. Figure 4(a) and (b) shows the boxplots of localization precision distribution in *x*-direction and *y*-direction of each group, respectively. As shown in Figure 4(a), *x*-direction localization precision using molecules of different $z_{1}$ -score groups were inversely correlative to $z_{1}$ -scores. Similar correlation was observed between *y*-direction localization precision and $z_{1}$ -scores (Figure 4(b)). These findings showed that stochastic motion of immobilized biomolecules deteriorates localization precision.

### 2.4 Localization precision can be improved by restricting confined stochastic motion

Increasing biomolecule size would increase the range of stochastic motion, leading to worse localization precision. To confirm this, we enlarge the oligonucleotide molecule from 40 nt to 60 nt. The histogram of localizations in x-and *y*-direction from 2119 molecules with 60 nt size (green line with circle markers) were showed in Figure 4(c) and (d), respectively, in comparison of those from molecules with 40 nt size (black line with diamond markers). The histogram data were normalized for better comparison. We can see that, the histogram curves of 60 nt molecules distribute wider than those of 40 nt molecules, suggesting worse localization precision.

Adding a “post-fix” step in sample preparation can minimize the bio-molecule motion. To test this, the localization precision of 8840 molecules with 40 nt size with paraformaldehyde cross-linking post fixation (red line with cross makers) were also calculated in Figure 4(c) and (d), both of which show the distribution narrower than the curves without post fix, suggesting improved localization precision.

When comparing molecules of similar z-scores from different samples, we found that in regard to each certain z-score range the localization precisions using 40 nt molecules (black) are all better than those using 60-nt molecules (green, Figure 4(e) and (f)) but not as good as those using samples with post fixation (red, Figure 4(e) and (f)). Therefore, these results reveal the impact of biomolecule stochastic motion on increasing localization uncertainty, providing potential solution for high resolution via reducing probe sizes or further constraining probes.

To further demonstrate improvement of localization precision by minimizing stochastic motion on biology samples, we performed immunofluorescence imaging of microtubules. Two microtubule samples were incubated with mouse antitubulin antibody (sigma) and then with AF647-conjugated antimouse secondary antibody, with labeling density low enough to ensure that localizations from individual molecules can be identified. One of the samples was post-fixed with 4% paraformaldehyde. The histogram of all the localizations from single molecules from the two samples were Gaussian fitted and plotted in Figure 4(g) (*x*-direction) and (h) (*y*-direction). We can see that the treatment of post fix (red solid line with circle markers) obviously narrowed down the distribution, in comparison of that without post fix (black dashed line with cross markers).

## 3 Conclusion and discussion

In this study, we have systematically assessed the confined stochastic motion of immobilized biomolecules in SMLM by calculating their $z_{1}$ -scores. Theoretically, the $z_{1}$ -scores are expected to be around zero for immobile molecules but to be negative for molecules undergoing any movements. Our analysis showed that $z_{1}$ -scores are shifting to around −1.1 from −2.7 after drift correction using fluorescent beads that are fixed to the gelatin-coated surface. Thermal motion of these fixed beads [21] contributes very little to the negative $z_{1}$ -scores (−1.1) after drift correction, which is indicated by comparing the $z_{1}$ -scores of 40 nt oligonucleotides corrected by less mobile beads to those corrected by more mobile beads (pair test, *p* value > 0.05). In addition, field-dependent aberrations such as field curvature and distortion might result in localization inaccuracy, which gets more severe the further away from the central of the field-of-view [22], [23], [24]. Interestingly, $z_{1}$ -scores obtained after drift correction with beads relatively distant from the central do not show a more negative trend, compared to those corrected with beads close to the center (pair test, *p* value > 0.05). In this regard, the negative $z_{1}$ -scores in our experiments, in which all fields are corrected with at least two different beads, are contributed largely by biomolecule stochastic motion rather than by field-dependent aberrations. Therefore, for the first time, we revealed that confined stochastic motion of biomolecules around their immobilized sites can be detected in dSTORM and worsened localization precision. These lines of evidence fill the gap in our knowledge about the detectability and impact of biomolecule stochastic motion in SMLM. Furthermore, we showed that localization precision could be deteriorated or improved via increasing biomolecule sizes or adding a post crosslinking step at the end of sample preparation, which reinforces or minimizes biomolecule stochastic motion, respectively. Taken together, these findings not only provide new insights into confined biomolecules stochastic motion but also offer practical strategies for improving localization precision. Similar phenomena and impacts are expected in PALM as both STORM and PALM provide unmatched super-resolution via sequentially resolving single molecules and requiring a relatively long acquisition time [8].

For super-resolution visualization of cellular ultrastructures, endogenous large bio-molecules like proteins or nucleic acids are commonly linked to photoswitchable fluorescent dyes via binding to specific antibodies or oligonucleotide probes for dSTORM imaging [2, 9], while PALM has been used to resolve proteins that are genetically coded to be fused to photoswitchable fluorescent proteins [10, 11]. In addition DNA-PAINT acts a newly-developed SMLM approach and provides superresolution via dynamically binding of fluorophore-conjugated short oligonucleotide (imager strand) to its complementary sequence (docking strand), which is usually linked to antibodies or oligonucleotide probes [25], [26], [27]. Biological samples for these three approaches involve two classes of biomolecules; molecules expressed by cells and extrinsic molecules for labeling the targets. In regard to sizes of the first class biomolecules, proteins expressed in cells generally exhibit molecular weights of a few to hundreds of Kilodaltons, which are corresponding to a few to tens of nanometers in sizes [28–30], whereas endogenous RNA and genomic DNA probably demonstrate relatively large sizes and form different configurations like long chains or networks that contain relatively independent nanoscale segments/domains [31]. These proteins or nucleic acids bind to surrounding molecules via different segments (domains) rather than the entire molecule surface. As a result, the cross-linking preimmobilization at the beginning of sample preparation fixes the associations of these molecules with surrounding molecular contexts to prevent diffusion and maintain their intracellular positions. In light of their nanometer sizes, a large amount of these biomolecules or their segments (domains) are expected to undergo confined stochastic motion in the imaging solution, since the preimmobilization probably fixes only those segments directly binding to the environmental context and leaves other parts stochastically moving around. In regard to sizes of the second class biomolecules, antibodies used in STORM or DNA-PAINT samples bind to target molecules via noncovalent association and exhibit sizes of 12–15 nm for primary antibody alone and sizes of 24–30 nm when primary antibodies are followed by secondary antibodies. Oligonucleotide probes for super-resolution visualization of endogenous DNA or RNA in previous reports contain tens of nucleotides that are extending without binding to any surrounding context for STORM or DNA-PAINT imaging [26]. In this study, sparsely immobilized oligonucleotides containing 40 or 60 nucleotides are exploited to mimic the confined stochastic motion of the aforementioned immobilized biomolecules in regular biological samples for SMLM, as these oligonucleotides are exhibiting nanoscale sizes within the size ranges of regular biomolecules and constrained at the fixed sites with partial sections of them free for stochastic motion. Despite different robustness for oligonucleotides, antibodies or proteins expressed by cells, experiments using oligonucleotides or antibodies agree well with each other that the post crosslinking step to minimize stochastic motion enhances the localization precision. These results indicate that the detectability and impact of confined stochastic motion observed for the immobilized single oligonucleotide samples in this study are applicable to biomolecules in regular biological samples for SMLM.

This study shows localization precision can be enhanced by limiting confined biomolecule stochastic motion. Adding an extra “post-fix” step, formaldehyde crosslinking at the end of sample preparation, has been shown to improve localization precision in dSTORM imaging of immobilized single-oligonucleotide samples as well as the regular cell samples stained for endogenous proteins. In this regard, we suspect that post crosslinking may affect SMLM samples in various aspects, leading to improved localization precision. Firstly, it can reinforce the intrinsic molecule immobilization, which is originally implemented by the preimmobilization at the beginning and more or less eliminated by the washing steps in sample preparation. As a result, those motions caused by unstably-immobilized intrinsic targets and causing a blurring effect can be largely avoided by such an extra step. Secondly, many cellular objects for SMLM imaging are commonly labeled with high densities of linkers including oligonucleotide probes, antibodies or fluorescent proteins. The final image of the labeled object is the superposition of images of each conjugated fluorophore on the linkers along the acquisition process. The post crosslinking probably results in covalently linking between closely-residing segments of linkers and thus restricts the range of their stochastic motion, despite the possibility that post crosslinking might pin a certain fluorophore to a position far away from the object. Stochastic motion of each fluorophore causes a wider distribution of its localizations and increases the probability of a more blurring image for a labeled cellular object; vice versa, restricting bio-molecule stochastic motion narrows down the localization distribution for each fluorophore, possibly leading to improved resolution. Thirdly, there is a possibility that post crosslinking might restrict fluorophore rotational motion, which might lead to localization inaccuracies according to the previous stimulation study [32]. To minimize the possibility of restricting fluorophore rotational motion, the most critical point is to avoid direct and rigid fixation of fluorophore molecules to the surrounding molecular context. In this regard, AF647 is a better choice than Cy5 for samples subjected to post crosslinking, since formaldehyde used in post cross-linking functions via covalently linking closely-residing primary amines while AF647 contains no primary amine (-NH2). Given the very small sizes of switchable organic fluorophores like AF647, a shorter oligonucleotide (e.g. 9–10 nt sequence) is not expected to limit the fluorophore rotational motion. Compared to the “post fix” strategy, shorter oligonucleotide probes or direct labeling with fluorophore-conjugated primary antibody or nanoantibody instead of indirect labeling with primary and second antibodies is capable of decreasing linkage error as previously reported [33, 34] and improving localization precision according to this study. Therefore, shorter linkers could be the first choices when figuring out the potential approach for improving image quality. More potential strategies to reduce confined stochastic motion of target bio-molecules, such as imaging the samples at lower temperature, could be tested in future experiments to achieve better SMLM resolution. Taken together, this study demonstrates the existence and impact of confined molecule stochastic motion in SMLM and provides new direction for enhancing localization precision in SMLM.

## 4 Materials and methods

### 4.1 dSTORM imaging system

The dSTORM system used in this study is based on an inverted optical microscope (IX-71, Olympus) with a 100× oil immersion objective lens (Olympus) as previously described [35]. A 641 nm laser (CUBE 640–100C;Coherent) is used to excite fluorescence and switch AF647 to the dark state. The illumination uses the highly inclined and laminated optical sheet (HILO) configuration [36]. The laser power densities used this study is approximately 1.45 kW/cm^2^ for the 641 nm laser unless otherwise indicated. A dichroic mirror (ZT647rdc, Chroma) is used to separate the fluorescence from the laser and a band-pass filter (FF01-676/37, Semrock) on the imaging path is used to filter the fluorescence. Raw images of the fluorescent signals in each nuclear field are acquired with an EMCCD (DU-897U-CV0, Andor) at 33 Hz for 8000 frames. To avoid focal drift, an antidrift system is used to sustain the focal position within 10 nm during image processing [37].

### 4.2 Sample preparation

To prepare single-molecule samples with immobilized oligonucleotides, cover glasses were cleaned by sonication for 15–25 min in water, washed with Mili-Q water, and then coated with 0.1% gelatin at room temperature for 10 min. Fluorescent microspheres (F8810, Thermo Fisher) of 200 nm in sizes were fixed on the gelatin-coated glasses with 4% paraformaldehyde for around 10 min at room temperature, so that they can act as fiducial markers to correct sample drift in the *x*–*y* plane during image acquisition. After being washed in PBS for multiple times, the glasses with beads were incubated for 30 min with 1 μM oligonucleotides, each of which was conjugated to an AF647 at its 5′ end and to biotin at its 3′ end, to allow for nonspecific association between biotin and gelatin, leading to immobilization of oligonucleotides sparsely on the glass surface. Subsequently, the samples were rinsed with PBS to remove unbound molecules, followed by treatment with or without 4% paraformaldehyde to provide samples with or without postfixation.

To prepare cellular samples sparsely stained for tubulin proteins for assessing the impacts of post-fixation on localization precision, mouse embryonic fibroblast (MEF) cells were fixed for 15 min at room temperature with buffer containing 3.7% formaldehyde, 0.3% Triton X-100, 0.1% Glutaraldehyde, 80 mM PIPES pH 6.8, 1 mM EGTA, and 1 mM MgCl_2_. The fixed cells were rinsed with PBS three times, incubated for 7 min with fresh prepare 1 mg/ml sodium borohydride to reduce background fluorescence, and washed with Mili-Q water for three times. Cell samples were blocked with 2% BSA-PBS for 30 min and incubated with mouse anti-tubulin antibody (sigma) in 2% BSA-PBS for 1∼2 h at room temperature, followed by AF647-conjugated anti-mouse secondary antibody for 1 h at room temperature. Subsequently, cell samples were treated with or without 4% paraformaldehyde and washed with PBS three times before imaging.

### 4.3 Image collection and processing

Samples on coverslips were embedded in dSTORM imaging buffer containing 50 mM Tris (pH 8.0), 10 mM NaCl, 1% *β*-mercaptoethanol (v/v), 10% glucose (w/v), 0.5 mg/mL glucose oxidase (G2133, Sigma), and 40 ug/mL catalase (C30, Sigma) [35, 38, 39]. Immediately after embedding, different samples of the same experiment set were subjected to dSTORM imaging field by field in turn to avoid any artificial difference caused by experiment condition changes with imaging time. For raw image analysis, a plugin Thunderstorm for Image J was applied. For cell experiment, the precise localization data were from point-like objects, each of which appeared as a cluster of multiple localizations in the final images and were away from any discernable microtubule filaments.

### 4.4 Simulation of confined biomolecule stochastic motion

Simulation of a 2D stochastic motion of 200 particles within a domain restricted by a radius R was performed. The radius is set as 14.6 nm, which approximates the length of the 40 nt oligonucleotide used in our experiment in Figure 3(a). The position was measured with a constant time interval Δt=30 ms for 8000 frames. The initial position is supposed to be (0, 0). The increment of position for each step is independent and follows a normal distribution, as described by

(8) $$\Delta_{x}=\Delta_{typ}\cdot randn\left(1\right)$$

(9) $$\Delta_{y}=\Delta_{typ}\cdot randn\left(1\right)$$

Here, $\Delta_{typ}$ is the typical movement distance of a molecule during the time interval Δt . The randn(1) generates a random number from the standard normal distribution.

The position recorded at each frame can be calculated from their increments,

(10) $$x_{n}=x_{n-1}+\Delta_{xn}$$

(11) $$y_{n}=y_{n-1}+\Delta_{yn}$$

Positions outside the circular domain will be discarded and regenerated by Eqs. (8)–(11).

We assume that the only effect the oligonucleotide has on the dye molecule is to exert a restoring force that increases linearly with the distance of the bead from the attachment point and is directed towards the attachment point. We model the force as a simple spring force $\vec{f}=-k\vec{r}$ . The spring force moves the dye molecule towards to the center with a speed $\vec{\nu }=\vec{f}/\zeta$ , where ζ is the dye molecule’s friction constant in imaging buffer. Then we can get the position of the dye molecule by superimposing an additional term $\vec{\nu }\Delta t=-k\vec{r}\Delta t/\zeta$ on the random steps of Eqs. (10) and (11),

(12) $$x_{n}=x_{n-1}+\Delta_{xn}-\left(k\Delta t/\zeta \right)x_{n-1}$$

(13) $$y_{n}=y_{n-1}+\Delta_{yn}-\left(k\Delta t/\zeta \right)y_{n-1}$$

To further considering the localization error, we assume a static localization precision $\delta_{static}$ . The localizations were calculated from Eqs. (12) and (13) as,

(14) $$x_{n}=x_{n-1}+\Delta_{xn}-\left(k\Delta t/\zeta \right)x_{n-1}+\delta_{\text{static}}\cdot \text{randn}\left(1\right)$$

(15) $$y_{n}=y_{n-1}+\Delta_{yn}-\left(k\Delta t/\zeta \right)y_{n-1}+\delta_{\text{static}}\cdot \text{randn}\left(1\right)$$

To simulate the sparse activation of AF647, we extracted the on/off pattern of frame sequence of 2073 molecules from our experiment. In our simulation of 2000 molecules, the on/off pattern of each molecule was randomly selected from the experimental data. Equations (12) and (15) were revised to

(16) $$x_{n}=\left[x_{n-1}+\Delta_{xn}-\left(k\Delta t/\zeta \right)x_{n-1}+\delta_{\text{static}}\cdot \text{randn}\left(1\right)\right]\cdot P_{n}$$

(17) $$y_{n}=\left[y_{n-1}+\Delta_{yn}-\left(k\Delta t/\zeta \right)y_{n-1}+\delta_{\text{static}}\cdot \text{randn}\left(1\right)\right]\cdot P_{n}$$

with $P_{n}=1$ standing for on-state and $P_{n}=0$ for off-state.

Equations (16) and (17) generate localizations for an individual molecule undergoing confined stochastic motion. Its $z_{1}$ -score and $z_{m}$ -score can be calculated as described in Section 2.2.

Three unknown parameters ( $\Delta_{typ}$ , $k^{\prime }=k\Delta t/\zeta$ , $\delta_{static}$ ) were chosen to ensure the output of simulation agree with the experimental data, generating best fit values of $\Delta_{typ}=5.1769\;nm$ , $k^{\prime }=0.1257$ and $\delta_{static}=5.5\;nm$ .

The simulated localization precision from 2000 molecules are 9.6348 nm in *x*-direction and 9.5143 nm in *y*-direction, in good agreement with our experimental localization precision of 2073 molecules (40 nt oligonucleotides with biotin-binding), which are 9.0365 nm in *x*-direction and 9.1096 nm in *y*-direction.

The simulated curve of $z_{m}$ -score with different frame is plotted in Figure 3(b). To reduce running time, 200 molecules are simulated in this part. The saturation behavior is in close analogy to that of experiment in Figure 3(a). The simulated curve starts with $z_{1}$ -score of −1.1 equal to that from experiment.

The outputs of localization precision, $z_{m}$ -score and $z_{1}$ -score match well with experimental data, suggesting the model work well.

### 4.5 Z-score rather than mean squared displacement for assessing information-incomplete molecule motion

In dSTORM imaging, the fluorophore conjugated to each single molecule is stochastically activated rather than continuously fluorescent, leading to incomplete molecule trajectory along the acquisition process. To assess the potential stochastic motion of biomolecules in dSTORM, it requires a certain method capable of revealing insights based on the incomplete trajectory information. Mean squared displacement (MSD) provides displacements of different positions of the same single molecule at different imaging time points and thereby is a common metric in tracking the motion of single particles that are continuously-illuminating during the image acquisition [40]. Z-score has been widely used for assessing significant difference of different samples, especially those with incomplete information or huge variations. To determine a method optimal for the current research goal, we compared the effects of MSD and z-score in analyzing the simulating position data of single molecules that are continuously or stochastically illuminating. Here, we simulated 500 molecules, which were undergoing stochastic motion with $\Delta_{typ}=5.1769\;nm$ as described by Section 4.4, during 1000 frames with the same frame interval (30 ms) as that has been adopted in dSTORM experiments in this study.

Firstly, we applied simulated data of continuously-illuminating molecules and compared the effects of both metrics in analyzing these data. The localizations of a single molecule were simulated as their position in Eqs. (12) and (13) without considering any localization uncertainty. The mean squared displacement as a function of frame interval ( i ) can be calculated as,

(18) $$MSD\left(i\right)=<{\left(x_{n+i}-x_{n}\right)}^{2}+{\left(y_{n+i}-y_{n}\right)}^{2}>$$

in which <> represents the average of all available displacements of a given duration of i frames. Supplemental Figure 1(a) and (b) display the MSD curve and $z_{m}$ -score curve which is ensemble-averaged of 28 molecules with the error bars. As shown in Supplemental Figure 1(a) and (b), MSD (a) and $z_{m}$ -score (b) can both be used to analyze stochastic motion of continuously illuminating single molecules/particles.

Then, we applied simulated data of molecules that were stochastically illuminating as those molecules in our dSTORM did. As shown in Supplemental Figure 1(c) and (d), the error bars of each data point are remarkably smaller in z-score curve (d) than those in MSD curve (c), and significant difference between neighboring data points at early times can be easier to be observed in z-score evaluation (d) than MSD analysis (c), indicating z score as an optimal choice for analyzing molecule motions with incomplete trajectories. In regard to the relatively large variations and uncertainty of experimental data compared to the simulated data, we adopt z-score rather than MSD for assessing the stochastically-illuminating biomolecules and identifying their potential stochastic motion in dSTORM imaging.

## Supplementary Material

Supplementary Material
