# Supplementary material for: Improved localization precision via restricting confined biomolecule stochastic motion in single-molecule localization microscopy
Source: Nanophotonics. 2021 Nov 16;11(1):53–65. doi: 10.1515/nanoph-2021-0481 (PMC11501310; doi:10.1515/nanoph-2021-0481)
Supplement: Supplementary file 1 — Supplementary Material [file j_nanoph-2021-0481_suppl.docx]

Supplementary Material


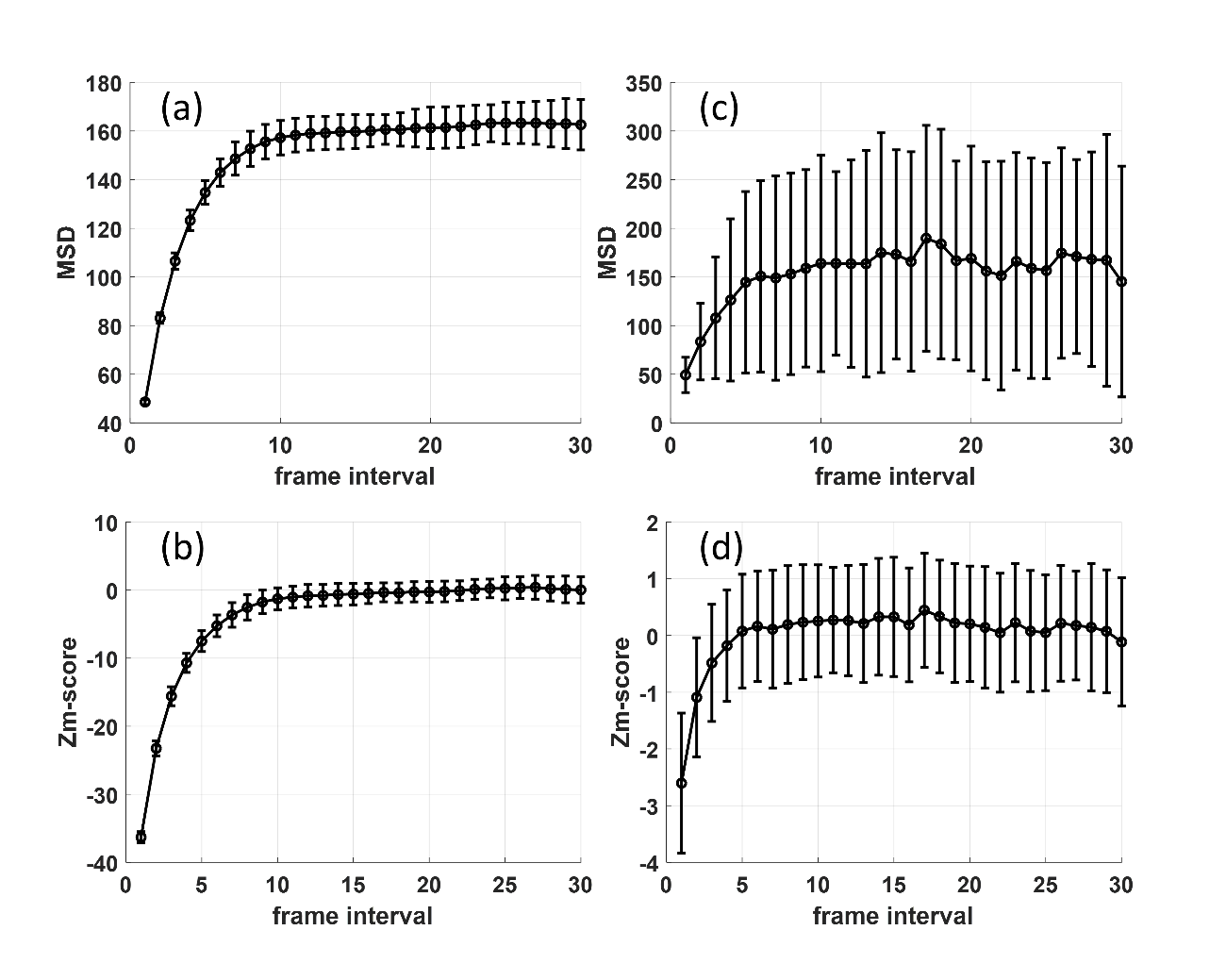


**Supplemental Figure 1. Comparison of the mean squared displacements (MSD) method (a, c) and the z-score method (b, d) in analyzing trajectory of continuously illuminating (a-b) or stochastically illuminating (c-d) molecules.** In (a and b), each circle represents the ensemble average of the calculated MSD or $z_{m}$-score of 28 continuously-illuminating molecules at a certain frame interval, with ensemble standard deviation (SD) as error bar. In such a continuously illuminating case, the MSD and $z_{m}$-score curves both show saturation patterns with small error bars. In contrast, with the application of 500 molecules are simulated to be stochastically excited as they do in SMLM, the MSD curve (c) shows large error bars, indicating low accuracy, whereas the $z_{m}$-score curve (d) shows relatively small error bars and reveals significant difference between data points at early times.
